# CircRNA_ACAP2 Suppresses EMT in Head and Neck Squamous Cell Carcinoma by Targeting the miR-21-5p/STAT3 Signaling Axis

**DOI:** 10.3389/fonc.2020.583682

**Published:** 2020-12-11

**Authors:** Chuan Ma, Tingting Shi, Zhuli Qu, Aobo Zhang, Zuping Wu, Huaqiang Zhao, Haoming Zhao, Hongyu Chen

**Affiliations:** ^1^ Department of Oral and Maxillofacial Surgery, School and Hospital of Stomatology, Cheeloo College of Medicine, Shandong University, Jinan, China; ^2^ Shandong Key Laboratory of Oral Tissue Regeneration and Shandong Engineering Laboratory for Dental Materials and Oral Tissue Regeneration, Jinan, China; ^3^ Department of Stomatology, Shandong Medical College, Jinan, China; ^4^ State Key Laboratory of Oral Diseases, West China Hospital of Stomatology, Sichuan University, Chengdu, China; ^5^ Department of Emergency School and Hospital of Stomatology, Cheeloo College of Medicine, Shandong University, Jinan, China

**Keywords:** HNSCC, microRNA, circRNA, STAT3, Zeb1

## Abstract

Circular RNAs (circRNAs) contain microRNA (miRNA)-specific binding sites and can function as miRNA sponges to regulate gene expression by suppressing the inhibitory effect of miRNAs on their target genes. MiR-21-5p has been reported to be involved in the development of head and neck squamous cell carcinoma (HNSCC) and plays an important role in the activation of epithelial-mesenchymal transition (EMT). However, the upstream regulatory mechanism and downstream targets of miR-21-5p in tumor cells remain unknown. CircRNA_ACAP2 inhibits the function of miR-21-5p by binding to its specific binding sites in HNSCC cells. Overexpression of CircRNA_ACAP2 inhibits the proliferation and migration of HNSCC cells, while downregulation of CircRNA_ACAP2 has the opposite effect. STAT3 is a direct target gene of miR-21-5p and a transcription factor of ZEB1. We demonstrate that CircRNA_ACAP2 functions as a tumor suppressor gene in HNSCC and that its function is regulated *via* the miR-21-5p/STAT3 signaling axis.

## Introduction

Head and neck squamous cell carcinoma (HNSCC) refers to an epithelial malignant tumor that originates in the head and neck, and over 600,000 new cases are reported every year (1, 2). The current treatment of head and neck cancer (HNC) patients is mainly based on the stage and anatomical location of the tumor, and therapies based on biological discoveries are rare. Although advances in technology and supportive treatment have improved the quality of life of HNC patients, the overall prognosis is still poor due to local recurrence and distant metastasis after surgery (3). Therefore, there is an urgent need to better understand the molecular mechanism of HNC progression to improve the prevention, diagnosis and personalized treatment of patients.

Circular RNAs (circRNAs) are an abundant and diverse class of noncoding RNA molecules that form a ring structure by covalent bonds and do not have a 5’ end cap or a 3’ end poly(A) tail (4, 5). The currently identified circRNAs are mainly derived from exons, but there are other types of circRNAs, such as intronic, intergenic, antisense, and sense overlapping (6, 7). MicroRNAs (miRNAs) are a group of evolutionarily conserved noncoding single-stranded RNAs consisting of 22–25 nucleotides that bind to the 3’ untranslated region (3’UTR) of target mRNA to repress gene expression (8, 9). As regulatory factors, miRNAs regulate physiological and pathological processes by blocking protein translation or inducing mRNA degradation to inhibit target gene expression and are widely involved in many biological processes, such as cell metabolism, proliferation, differentiation and apoptosis (10, 11).

In a previous study, CircRNA_ACAP2 was identified as a therapeutic target for the treatment of colon cancer (10). However, the mechanism of action of CircRNA_ACAP2 in HNSCC progression remains unknown. We determined the expression levels of miR-21-5p and CircRNA_ACAP2. The linear isomers of CircRNA_ACAP2 in HNSCC tissues were used to determine its expression pattern. Moreover, we overexpressed CircRNA_ACAP2 in HNSCC cell lines and performed cell proliferation experiments to elucidate its function. We demonstrated that the CircRNA-ACAP2/miR-21-5p/STAT3 regulatory feedback loop could affect the epithelial-mesenchymal transition (EMT) of HNSCC cells. By exploring this hypothesis, we may gain insights into the pathogenicity of HNC and provide new therapeutic targets for the treatment of HNSCC.

## Materials and Methods

### Patients

A total of 102 patients diagnosed with HNSCC were included in this study. All fresh tissues were collected between July 2011 and Dec 2015 during radical surgery at the Department of Oral and Maxillofacial Surgery, School and Hospital of Stomatology, Cheeloo College of Medicine, Shandong University. The samples were frozen in liquid nitrogen for 5 min and stored at −80°C. None of the enrolled patients received chemotherapy, radiotherapy, or targeted therapy before radical surgery. All patients were followed up for over 5 years or until Dec 2019. The study protocol was approved by the Institutional Review Board of School and Hospital of Stomatology, Cheeloo College of Medicine, Shandong University. Informed consent was obtained from all patients involved in this study. All methods were performed in accordance with the relevant guidelines and regulations.

### Immunohistochemistry

Excised tumor and adjacent tissues were fixed in 4% paraformaldehyde, dehydrated, paraffin-embedded, and cut into sections. Consecutive 4-μm-thick sections were analyzed using primary antibodies against ZEB1 (human; ab181451; 1:200; Abcam), phospho-STAT3 (human; #9145; 1:100; Cell Signaling Technology), STAT3 (human; #4695; 1:100; Cell Signaling Technology), TWIST-1(human; PA5-49688; 1:100; Invitrogen)and a biotin-conjugated goat anti-rabbit polyclonal antibody (1:50; ZSGB-BIO, Beijing, China) as the secondary antibody. Images were obtained by light microscopy (Olympus, Japan) at 100× and 400× magnification and quantified using Image-Pro Plus. Five random fields were examined per animal.

### Cell Culture

Human HNSCC cell lines, namely, HN-4, HN-9, HN-30, SCC-4, SCC-9, SCC-25, and CAL-27, were used in this study. HN-4, HN-9, HN-30, SCC-4, SCC-9, and SCC-25 cells were purchased from the Cell Bank of the Chinese Academy of Sciences (Shanghai, China), and CAL-27 cells were purchased from the American Type Culture Collection, Manassas, VA. These cells were cultured in Dulbecco’s modified Eagle’s medium (DMEM; Gibco-BRL, Grand Island, NY).

All cell lines were cultured in DMEM/F12 (1:1) medium (Gibco-BRL). The media were supplemented with 10% heat-inactivated fetal bovine serum (FBS) (Gibco-BRL), penicillin (100 units/ml), and streptomycin (100 μg/ml). The cells were cultured at 37°C in a humidified 5% CO_2_ atmosphere.

### miRNA-21-5p-Mimic Transfection

The miR-21-5p mimic and its negative control (NC) were synthesized by RiboBio (Guangzhou, China). Wild-type cells (5.0×10^5^ cells/well) were grown in 6-well plates in 2 ml culture medium. When cell confluence reached 50%–60%, the miR-21-5p mimic and its negative control were treated with Lipofectamine 2000 reagent (Invitrogen, USA) according to the manufacturer’s instructions. The cells were transfected in Opti-MEM (Gibco, USA) for 8 h, and then the medium was changed to normal culture medium. After 48 h, the cells were harvested for western blot and qRT-PCR. All groups were plated in 6-well culture plates at the same time and were harvested 48 h later.

### Lentiviral Transduction and Screening of Stable Strains

The CircRNA-ACAP2 and miR-21-5p lentiviral expression vectors (wild-type and mutant) were constructed by GeneChem Biotechnology Co., Ltd. The CircRNA-ACAP2 lentiviral expression vector (CircRNA-ACAP2 vector) conferred puromycin resistance, while the miR-21-5p lentiviral expression vector (miR-21-5p vector) was C-terminally tagged with an HA epitope and conferred blasticidin resistance. Lentiviral transduction was performed following the manufacturer’s instructions. After 72 h of transfection, the culture medium was mixed with puromycin/blasticidin at a final concentration of 3–10 μg/ml. After culturing with puromycin/blasticidin and passaging 2–3 times, stably transduced cells were screened.

### Wound Healing Assay

Tumor cells were plated in 6-well plates, transfected or pre-treated as indicated, and cultured to confluency. Cells were serum-starved and scraped with a P200 tip (time 0), washed with PBS, and incubated with serum-free DMEM. Pictures of five non-overlapping fields were taken at 24 h.

### Cell Proliferation Assay

Cells transfected for 24 h with miRNA mimic or stably transduced cells were seeded into 96-well plates at a density of 1000 cells per well in triplicate. The cells were harvested, and 10 μl of CCK-8 reagent (Dojindo, Kumamoto, Japan) was added to 100 μl of culture medium. The cells were subsequently incubated for 2 h at 37°C, and the optical density was measured at 450 nm using a microplate reader (SpectraMax i3, Molecular Devices, USA).

### Reverse Transcription-Quantitative Polymerase Chain Reaction Analysis

For RNA extraction, HNSCC tissues were harvested and cut into small pieces (< 2 × 2 mm^2^). The pieces were immediately frozen in liquid nitrogen and then ground into powder. Total RNA was extracted using TRIzol reagent (Invitrogen) according to the manufacturer’s protocol. For RT-PCR, we used the SYBR Two Step Reverse Transcription-Quantitative Polymerase Chain Reaction (RT-qPCR) Kit (TaKaRa, USA, catalog number RR037A) following the manufacturer’s instructions. The cycles were as follows: initial denaturation at 95°C for 30 s, followed by 40 amplification cycles of 95°C for 5 s and 60°C for 30 s. Values were quantified using the comparative cycle threshold method, and samples were normalized to GAPDH. Quantification of the relative expression levels was performed by the 2^-ΔΔCT^ method.

### Western Blotting

Cell and tissue lysates were prepared using modified RIPA buffer. Total protein concentrations were determined by a bicinchoninic acid (BCA) protein assay kit (Beyotime, catalog number P0012). Protein solution (approximately 15 μl) was resolved by 10% SDS-polyacrylamide gel electrophoresis and transferred to a polyvinylidene difluoride (PVDF) membrane (Millipore, catalog number IPVH00010). PVDF membranes containing the protein were blocked in 5% nonfat milk at room temperature for 2 h, and then the membranes were incubated with the following primary antibodies: anti-ZEB1 (human; ab181451; 1:1,000; Abcam), anti-phospho-STAT3 (human; #9145; 1:1,000; Cell Signaling Technology), and anti-STAT3 (human; #4695; 1:1,000; Cell Signaling Technology). All primary antibodies were diluted with 5% TBST buffer (with 0.1% Tween 20) and incubated overnight at 4°C. After washing with TBST buffer (with 0.1% Tween 20) three times, the membranes were incubated for 2 h with secondary horseradish peroxidase-conjugated anti-mouse (1:10,000, Servicebio, catalog number GB23301) and anti-rabbit (1:10,000, Servicebio, catalog number GB23303) antibodies. After the membranes were washed with TBST, the target proteins on the membrane were detected by an ECL detection system (Millipore, catalog MA01821). Protein band images were digitally captured, and the intensity of the bands (pixels/band) was determined using ImageJ densitometry analysis software in arbitrary optical density units.

### Fluorescence *In Situ* Hybridization Assay

Fluorescence-labeled probes for CricRNA-ACAP2, miR-21-5p were designed and synthesized, and fluorescence *in situ* hybridization (FISH) experiments were performed using a Ribo™ Fluorescent *In Situ* Hybridization kit (RiboBio). Images were acquired on a TCS SP2 laser-scanning confocal microscope (Leica Microsystems, Germany).

### Target Prediction

TargetScan and miRanda were used to predict potential CircRNA_ACAP2 targets. Only one target miRNA with expression in human tissues was studied further. The target genes of miR-21-5p were STAT3, GDF-5, STAT3, TIMP-3, and Smed2/3, and STAT3 was the most differentially expressed in cancer tissues and noncancerous tissues. The 3′UTR of STAT3 mRNA possesses a putative miR-21-5p binding site.

### Luciferase Reporter Assay

A dual-luciferase reporter assay was performed to validate the target relationship. A CircRNA_ACAP2 segment (112 bp) with either a mutant or wild-type seed region was synthesized, cloned into the psiCHECK-2 vector (Applied Biosystems, USA), and inserted into the pmirGLO Dual-Luciferase Vector (Promega, catalog number E133A). All cell lines were transfected using Lipofectamine 2000 (Invitrogen, USA). The cells (1×10^5^ cells/well) were transiently transfected with the CircRNA_ACAP2 segment vector. To knock down miR-21-5p expression, siRNAs against miR-21-5p and a negative control siRNA (NC-siRNA) were prepared (GeneChem, Shanghai, China). Cotransfection of 20 nmol/L miR-21-5p mimics or control was then performed. After 48 h, HN-9 and SCC-25 cells were harvested, and luciferase activity was assayed using the Dual-Luciferase Reporter Assay System (Promega, catalog number E1910).

### Statistical Analysis

The statistical analyses were performed using GraphPad Prism software (GraphPad Software Inc.). For data with normal distribution and/or equal variances, significant differences between two and more than three groups were determined by two-tailed Student’s t test and one-way ANOVA followed by Bonferroni’s *post hoc* comparison test, respectively. P values lower than 0.05 were considered to be statistically significant. The results are presented as the mean ± SEM from at least three independent experiments.

## Results

### CircRNA-ACAP2 Is Downregulated in HNSCC Tissues, and miR-21-5p, P-STAT3 Are Upregulated and Negatively Correlated

**Figure 1:**
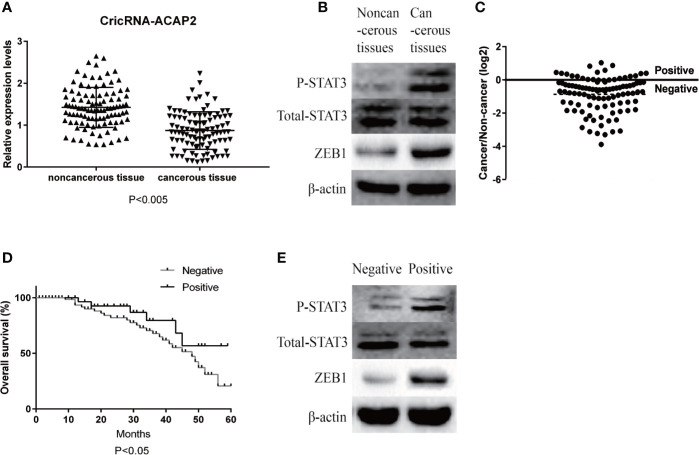
Relative expression level of CricRNA-ACAP2. **(A)** Relative expression level of CricRNA-ACAP2 in 102 head and neck squamous cell carcinoma (HNSCC) tissues and in paired adjacent normal tissues. **(B)** Relative protein expression level of STAT3,P-STAT3,ZEB1 in HNSCC tissues and in paired adjacent normal tissues. **(C)** Cancer/non-cancer ratio of CricRNA-ACAP2 expression level in 102 HNSCC tissues and in paired adjacent normal tissues. **(D)** The Kaplan‐Meier survival analysis indicated that negative group has a worse overall survival compared to the positive group. **(E)** Relative protein expression level of STAT3,P-STAT3,ZEB1 in negative HNSCC tissues and in positive HNSCC tissues. Error bars represent mean ± standard deviation (SD). P value was written in the figure.

We investigated 102 pairs of human HNSCC specimens and adjacent noncancerous tissue samples *via* RT-qPCR to confirm CircRNA_ACAP2 expression in HNSCC. CircRNA_ACAP2 expression was markedly downregulated in 89.2% (91/102) of HNSCC tissues (**Figure 1A**) and was significantly lower than that in the corresponding adjacent noncancerous tissues (P < 0.005). We examined the expression of P-STAT3 and ZEB1 in cancer tissues and noncancerous tissues and found that the expression of ZEB1 in HNSCC tissue was significantly upregulated, while STAT3 was significantly phosphorylated (**Figure 1B**). In addition, we divided all the cases into two groups according to the ratio of CircRNA_ACAP2 expression levels in cancer tissues and noncancerous tissues (**Figure 1C**). The results of Kaplan-Meier survival analysis indicated that the positive group had better overall survival than the negative group (**Figure 1D**). We compared the expression of P-STAT3 and ZEB1 at the protein level and found that the expression of ZEB1 at the protein level was significantly higher in the negative group than in the positive group, while the phosphorylation level of STAT3 was obviously higher in the negative group than in the positive group (**Figure 1E**).

**Figure 2:**
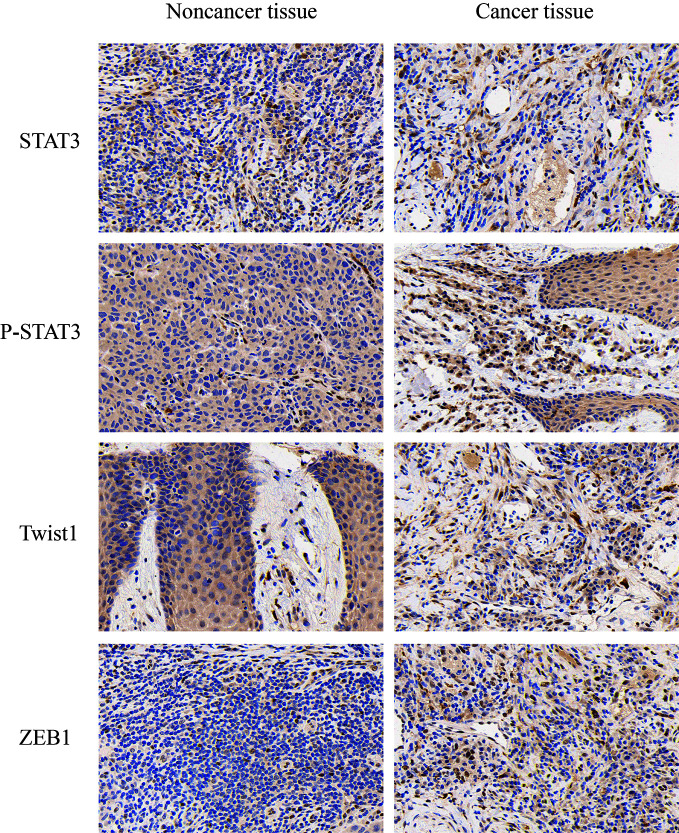
Immunohistochemistry. P-STAT3, ZEB1, and Twist1 in squamous cell carcinoma tissues and adjacent tissues were detected by immunohistochemistry. P value was written in the figure.

**Figure 3:**
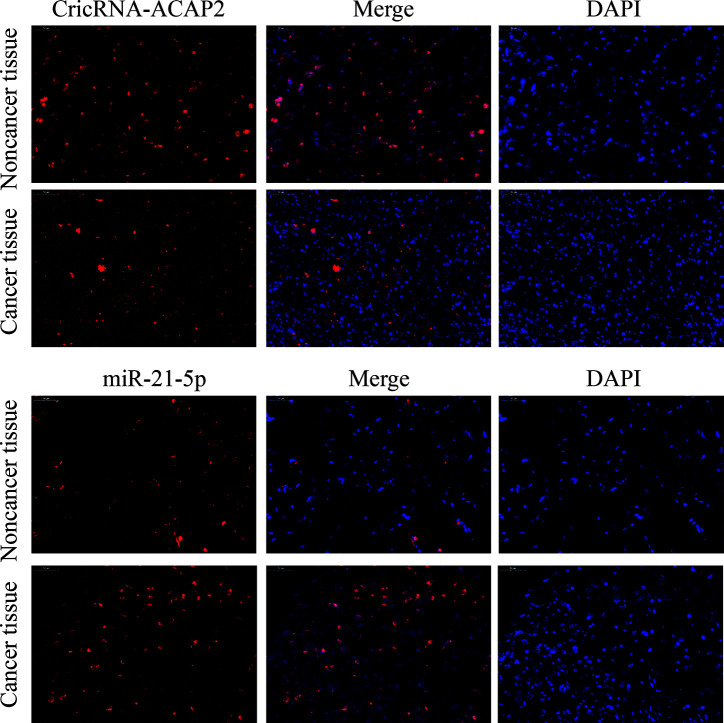
Fluorescence *in situ* hybridization (FISH) assay. FISH analysis of CricRNA-ACAP2 and miR-21-5p in HNSCC tissue. (The nuclei were stained with DAPI).

We detected P-STAT3, ZEB1, and Twist1 in squamous cell carcinoma tissues and adjacent tissues by immunohistochemistry. These proteins are all related to the epithelial-mesenchymal transition effect of tumor cells, and these proteins are expressed in cancer tissues. Higher than adjacent tissues. We selected the STAT3 protein as a control, and found that the total STAT3 expression did not change significantly (**Figure 2**). The expression of CricRNA-ACAP2 in non-cancerous tissues is higher than that in cancerous tissues, while the expression trend of miR-21 is opposite (**Figure 3**). The amount of CricRNA-ACAP2 in the cytoplasm was higher than that observed in the nucleus, revealing that CricRNA-ACAP2 is predominantly located in the cytoplasm.

### The Expression of CircRNA_ACAP2 and miR-21-5p in Various Head and Neck Squamous Cell Carcinoma Cell Lines

**Figure 4:**
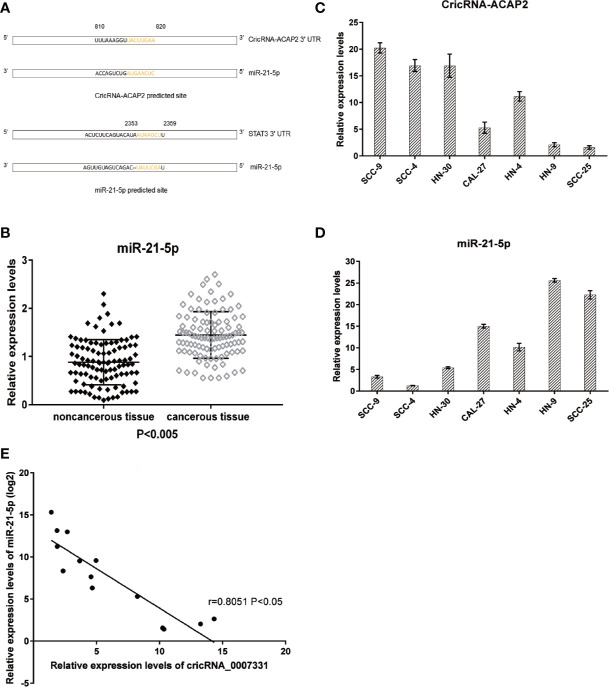
Predicted downstream miRNA and target gene. **(A)** Predicted binding site of downstream miRNA and target gene. **(B)** Relative expression level of miR-21-5p in 102 HNSCC tissues and in paired adjacent normal tissues. **(C, D)** Endogenous CricRNA-ACAP2 and miR-21-5p expression in serial HNSCC cell lines. **(E)** Correlation of expression between miR‐21-5p and CricRNA-ACAP2. Error bars represent mean ± standard deviation (SD). P value was written in the figure.

The potential targets of CircRNA_ACAP2 were searched in bioinformatics databases *via* TargetScan and miRanda to explore the underlying molecular mechanism. In addition, the potential binding sites of miR-21-5p in STAT3 were predicted (**Figure 4A**). We analyzed miR-21-5p expression in human HNSCC cell lines. The expression level of miR-21-5p in HNSCC tissues was significantly higher than that in adjacent tissues (P<0.005) (**Figure 4B**). To explore the relationship between CircRNA_ACAP2 and miR-21-5p in various HNSCC cells, we examined the expression levels of CircRNA-ACAP2 (**Figure 4C**) and miR-21-5p (**Figure 4D**) in different HNSCC cell lines, namely, HN-4, HN-9, HN-30, SCC-4, SCC-9, SCC-25, and CAL-27. A significant negative correlation was found between the expression levels of miR-21-5p and CircRNA-ACAP2 (**Figure 4E**).

### MiR-21-5p Is a Direct Target of CircRNA-ACAP2, and STAT3 Is a Direct Target Gene of miR-21-5p

**Figure 5:**
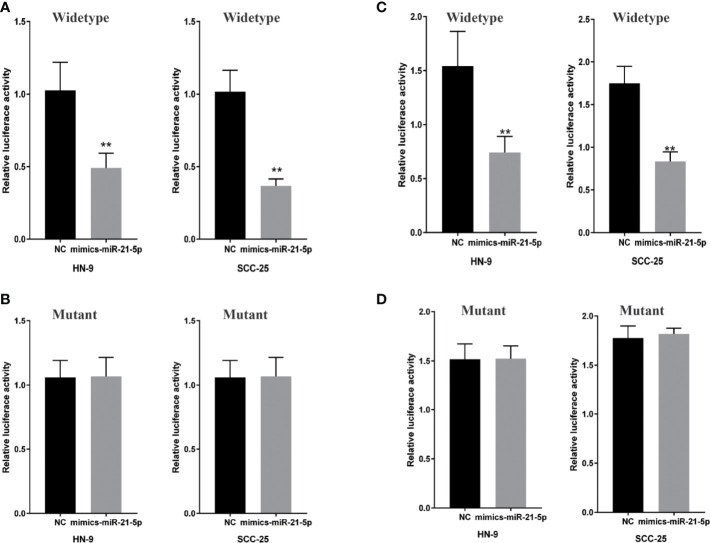
MiR-21-5p is a direct target of CricRNA-ACAP2 and P-STAT3 is a direct target gene of miR-21-5p. **(A)** Fluorescence intensity of CricRNA-ACAP2 segment was significantly reduced in the miR‐21-5p mimics and wild‐type dual‐luciferase reporter plasmid transfected group in the two HNSCC cell lines. **(B)** Fluorescence intensity of CricRNA-ACAP2 segment showed no significant change in the miR‐21-5p mimics and mutant dual‐luciferase reporter plasmid‐transfected group in the two HNSCC cell lines. **(C, D)** Dual luciferase reporter assay to validate target relationship between Spry-1 and miR-21-5p. HN-9 and SCC-25 were transfected with mimic miR-21-5p. Error bars represent mean of three separate determinations ± standard deviation (SD). Asterisk indicates statistically significant changes: * (P < 0.05), ** (P < 0.01).

To determine whether miR-21-5p directly targets CircRNA_ACAP2, we generated dual-luciferase reporter plasmids carrying a fragment of the mutant or wild-type CircRNA_ACAP2 sequence and the predicted miR-21-5p binding site. A dual-luciferase reporter assay was then performed in randomly selected HN-9 and SCC-25 cells. The normalized fluorescence intensity of the wild-type CircRNA_ACAP2 reporter was considerably lower in cancer cells cotransfected with the miR-21-5p mimics than the control (**Figure 5A**). In contrast, no significant difference was found between the control cells and cells cotransfected with miR-21-5p mimics and the mutant CircRNA_ACAP2 reporter (**Figure 5B**).

A dual-luciferase reporter assay was conducted to investigate whether STAT3 mRNA is a target of miR-21-5p. HN-9 and SCC-25 cells were cotransfected with the miR-21-5p mimic and pmirGLO vectors, and a considerable reduction was observed in the STAT3 wild-type 3′UTR group relative to the negative control vector group, but no significant reduction was found in the STAT3 mutant 3′UTR group (**Figures 5C, D**). Collectively, the abovementioned results suggested that miR-21-5p could regulate STAT3 protein expression in chondrocytes by targeting the STAT3 mRNA 3′UTR.

### Different Effects of CircRNA-ACAP2 and miR-21-5p on the Proliferation of Head and Neck Squamous Cell Carcinoma Cells

**Figure 6:**
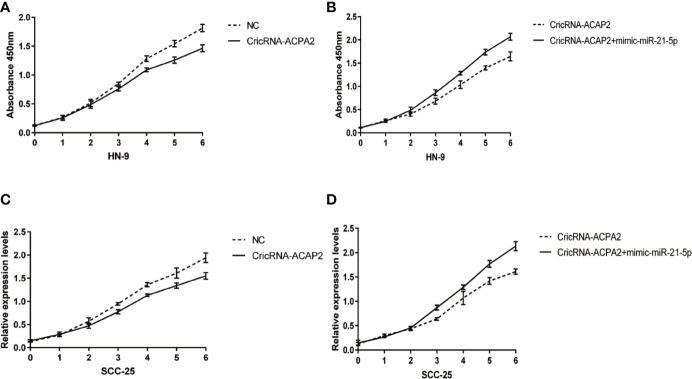
CricRNA-ACAP2‐miR‐21-5p axis mediated proliferation of head and neck squamous cell carcinoma (HNSCC) cell lines. **(A, C)** CricRNA-ACAP2 overexpression inhibits cell growth in HN-9 and SCC-25. **(B, D)** miR‐21-5p can interrupt the cell growth inhibition function of CricRNA-ACAP2.

**Figure 7:**
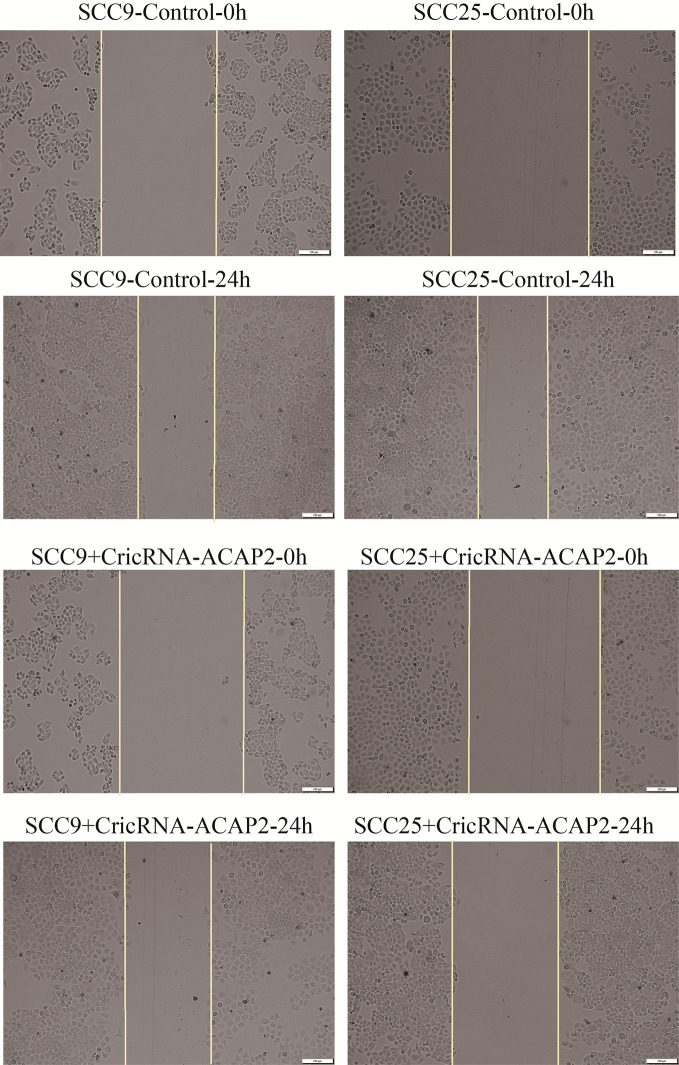
Wound healing assay. The results of Wound healing assay demonstrated that CircRNA_ACAP2 overexpression suppressed cell migration and invasion in SCC-25 cells, while CircRNA_ACAP2 overexpression has no effect on cell migration and invasion in SCC-9 cells.

We performed a functional assay by transfecting pcDNA3.1-CircRNA_ACAP2/miR-21-5p mimics or negative control reagents into HN-9 and SCC-25 cells, and the results showed that CircRNA_ACAP2 had low expression in all cell lines tested. We evaluated the effect of CircRNA_ACAP2 on the proliferation of HNSCC cells through the CCK-8 assay. Overexpression of CircRNA_ACAP2 inhibited the proliferation of HN-9 and SCC-25 cells (**Figures 6A, C**). Therefore, our results showed that CircRNA_ACAP2 inhibited the proliferation of HNSCC cells. If the effect of CircRNA_ACAP2 is specific, the effect of CircRNA_ACAP2 overexpression must be suppressed by coexpression of miR-21-5p. To test this hypothesis, miR-21-5p mimics (which increase miR-21-5p levels) were cotransfected with pcDNA3.1-CircRNA_ACAP2 into HN-9 and SCC-25 cells. Then, CCK-8 measurements were performed. In HN-9 and SCC-25 cells, cells coexpressing CircRNA_ACAP2 and miR-21-5p mimics had significantly increased growth rates compared to cells coexpressing CircRNA_ACAP2 and the control (**Figures 6B, D**). The results of Wound-healing assay demonstrated that CircRNA_ACAP2 overexpression suppressed cell migration and invasion in SCC-25 cells, while CircRNA_ACAP2 overexpression has no effect on cell migration and invasion in SCC-9 cells (**Figure 7**).

### CircRNA-ACAP2 and miR-21-5p Regulate Epithelial–Mesenchymal Transition Through STAT3

**Figure 8:**
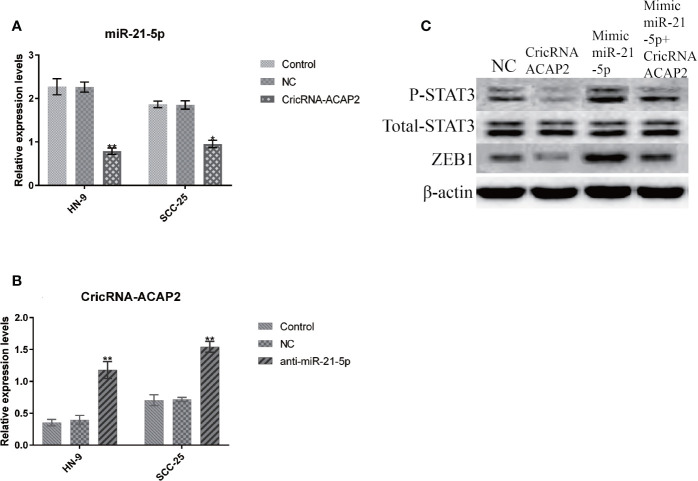
CricRNA-ACAP2 and miR-21-5p regulate ERK phosphorylation through P-STAT3. **(A)** The expression of miR-21-5p in HN-9 and SCC-25 is significantly suppressed *via* transfecting pcDNA3.1-CricRNA-ACAP2. **(B)** When miR-21-5p was knocked down by siRNA, the expression of CricRNA-ACAP2 was significantly up-regulated. **(C)** CricRNA-ACAP2 and miR-21-5p regulate ERK phosphorylation throughP-STAT3.

When SCC-25 cells were transfected with pcDNA3.1-CircRNA-ACAP2, the expression of miR-21-5p was significantly suppressed (**Figure 8A**). Conversely, when miR-21-5p was knocked down by siRNA, the expression of CircRNA-ACAP2 was significantly upregulated (**Figure 8B**). Simultaneously, in the pcDNA3.1-CircRNA-ACAP2 group, we found that the expression of ZEB1 was significantly upregulated and that STAT3 was phosphorylated, with no significant change in the expression of total STAT3. When cells were transfected with the miR-21-5p mimic, the expression of ZEB1 was significantly increased, and the phosphorylation of STAT3 was significantly upregulated. However, after cotransfection of CircRNA_ACAP2 and miR-21-5p mimics, the expression of ZEB1 was inhibited (**Figure 8C**).

## Discussion

CircRNAs are an intriguing class of RNA molecules due to their covalently closed structure, high stability, and implicated roles in gene regulation (12). The downregulation of circRNAs in proliferative cells across different tumor types could indicate that some circRNAs may have tumor-suppressive roles (13). Although circRNAs were discovered 40 years ago, they have become a hot spot in related disease research in recent years due to the increased understanding of their biological characteristics, production mechanism and functional significance (14). Most circRNAs are endogenous noncoding RNAs that have a high degree of conservation among species (7). Compared to linear RNAs, circRNAs have high stability. Some circRNAs have miRNA-specific binding sites (12, 15). Consequently, by competitively binding with miRNAs to inhibit the degradation of the corresponding target gene mRNAs, circRNAs can function similar to lncRNAs, which act as miRNA sponges to adsorb miRNAs and regulate the expression of downstream target genes (16, 17).

Due to the development of microarray chips and sequencing technology, a large number of studies have detected the abnormal expression of circRNAs in cancer samples (15). In pancreatic ductal adenocarcinoma (PDAC), the abnormal expression of circRNAs has been confirmed as one of the characteristics of early PDAC (18). circRNA_100855 and circRNA_104912 are the most significantly deregulated circRNAs in laryngeal cancer, while circRNA_001059 and circRNA_000167 are significantly inactivated in radiation-resistant esophageal cancer (19, 20). Many circRNAs with miRNA response elements (MREs) are found in basal cell carcinoma (BBC) and cutaneous squamous cell carcinoma (CSCC) (21, 22). More recently, it was reported that 69 differentially expressed circRNAs might interact with certain miRNAs to influence mRNA expression in HNSCC (23).

Through bioinformatics analysis, we found that hsa_circ-ACAP2 is upregulated in many types of tumor tissues. Hsa_circ_ACAP2 is encoded by the ACAP2 gene, which is a homolog of *C. elegans* CNT-1, a gene that promotes apoptosis, and has the same inositol phosphate-binding pattern as CNT-1. We predicted that hsa-miR-21-5p is a specific target of circRNA-ACAP2 through the bioinformatics tools TargetScan and miRanda (10). Moreover, some studies have found that miRNA-21-5p is highly related to STAT3 transcription, and it is also one of the few miRNAs that can promote STAT3 expression. Through comparisons and screening of human gene libraries, we know that the target genes of miRNA-21-5p include IL-6 receptor (IL-6R), STAT3, TIMP3 and so on (24). The JAK/STAT3 signaling pathway activated by the IL6/IL-6R/IL-6Rβ (gp130) complex plays a key role in the growth and development of many human cancers. The IL-6/JAK/STAT3 pathway is overactivated in many cancer patients, and numerous studies involving preclinical *in vitro* and *in vivo* models have shown that targeting a single nodule in this pathway can have antitumor effects. Therefore, therapies targeting this pathway may benefit cancer patients by inhibiting tumor cell growth and stimulating antitumor immunity. IL-6 levels are elevated in a large number of patients with malignant solid tumors (25). Elevated IL-6 levels stimulate excessive activation of the JAK/STAT3 signaling pathway, often predicting poor patient prognosis. Increased IL-6 levels have been observed in the serum and tumor microenvironment, and all immune cells (such as NK cells, effector T cells, and DCs) in the tumor microenvironment express IL-6R. The activated STAT3 signaling pathway initiates the transcription process, including the expression of PD-L1, which in turn enhances tumor immunosuppression. Therefore, the effects of inhibiting miR-21-5p will affect the tumor in two aspects in terms of inhibiting the expression of IL-6R and STAT3 (26, 27). Drugs targeting various lymph nodes, including IL-6, IL-6R, and JAK, have been approved by the FDA and can be used to treat inflammatory diseases or myeloproliferative tumors. At present, many such drugs are also under active research to treat other malignant tumors and solid tumors of the hematopoietic system (25).

EMT is a process in which polar epithelial cells transform into mesenchymal cells and acquire invasion and migration capabilities (28). Current studies have found that partial activation of EMT is the main driving force for tumors from initiation to metastasis (29). EMT is a multistep dynamic process, in which the connections between epithelial cells disappear, the tissue structure becomes loose, and cubic epithelial cells acquire a spindle-shaped fibrous cell morphology and exhibit aggressiveness (30). HNSCC cells with an EMT phenotype have strong motility, allowing them to infiltrate locally, invade blood vessels and lymphatic vessels, and migrate to target organs for secondary metastasis. After reaching the target organ, cancer cells can initiate mesenchymal epithelial transformation to rebuild intercellular connections and the cytoskeleton to form metastases. Epithelioid tumor cells have strong proliferation ability and help metastatic cancer cells complete metastatic colonization (31).

Our study showed that miR-21-5p is of great significance in the early diagnosis, treatment and prognosis of HNSCC. However, it is still difficult to use miR-21-5p as a therapeutic target. CircRNAs are more stable than miRNAs. We think that it can be used as a targeted drug for the treatment of HNSCC. In addition, IL-6R is one of the target genes of miR-21-5p, and secreted IL-6R (sIL-6R) is associated with metastasis and immunosuppression in most cancers. Furthermore, the JAK/STAT3 signaling pathway is regulated by the IL-6/IL-6R/gp130 complex. Epigenetic changes play an important role in the abnormal activation of the IL-6/IL-6R/JAK/STAT3 pathway in cancer, and changes in transcription factor expression and/or activation may be involved in cancer progression (25). MiR-21-5p can simultaneously target two signaling pathways mediated by IL-6 (32, 33). Circulating IL-6 levels have been verified to be a prognostic indicator of survival in several different types of cancers and a predictor of the response to treatment.

## Data Availability Statement

The raw data supporting the conclusions of this article will be made available by the authors, without undue reservation.

## Ethics Statements

The studies involving human participants were reviewed and approved by The Ethics Committee of Shandong University. The patients/participants provided their written informed consent to participate in this study. No animal studies are presented in this manuscript. No potentially identifiable human images or data is presented in this study.

## Author Contributions

Conceived and designed the experiments: CM, TS, HAZ; Performed the experiments: HC, ZQ, CM; Analyzed the data: AZ, ZW; Contributed reagents/materials/analysis tools: CM, TS, HUZ, HAZ. All authors contributed to the article and approved the submitted version.

## Funding

This work was supported by the Natural Science Foundation of Shandong Province (No. ZR2018PH022) and the China Postdoctoral Science Foundation (No. 2019M652408).

## Conflict of Interest

The authors declare that the research was conducted in the absence of any commercial or financial relationships that could be construed as a potential conflict of interest.
